# Data on length of parathyroidectomy surgery and intraoperative parathyroid hormone (PTH) assay turnaround times following a switch in the location for intraoperative PTH testing from near point-of-care to central laboratory

**DOI:** 10.1016/j.dib.2020.106252

**Published:** 2020-09-01

**Authors:** Denise Jacob, Anna E. Merrill, Dena R. Voss, Tami Bebber, Scott R. Davis, Jeff Kulhavy, Matthew D. Krasowski

**Affiliations:** Department of Pathology, University of Iowa Hospitals and Clinics, 200 Hawkins Drive, C-671 GH, Iowa City, IA 52242, USA

**Keywords:** Parathyroid hormone, Clinical chemistry tests, Parathyroidectomy, Intraoperative care, Clinical laboratory services

## Abstract

Intraoperative monitoring of parathyroid hormone (PTH) is commonly used during parathyroidectomies. There are a number of practical challenges in achieving rapid turnaround time (TAT) for intraoperative PTH testing, whether the testing is performed point-of-care, near point-of-care, or in a central clinical laboratory. In the related research article, we analyzed a decade of data from 3025 intraoperative PTH tests on 897 unique patients. Of these, 1787 tests on 514 unique patients (375 female, 139 male) occurred while intraoperative PTH measurement was done as near point-of-care testing; the remaining 1238 tests on 383 unique patients (282 female, 101 male) occurred after a switch to intraoperative PTH measurement by the hospital central laboratory. The data in this article provides the patient age, gender, location of surgery (main operating rooms vs. ambulatory surgery center), incision to close time for surgery, and operation start to end times. For the central laboratory testing, additional data are provided for the intraoperative PTH TAT. The analyzed data is provided in the supplementary tables included in this article. Plots of operation start and end times are also included. The dataset reported is related to the research article entitled “Evaluation of Switch from Satellite Laboratory to Central Laboratory for Testing of Intraoperative Parathyroid Hormone” [D. Jacob, G. Lal, D.R. Voss, T. Bebber, S.R. David, J. Kulhavy, S.L. Sugg, A.E. Merrill, M.D. Krasowski, Evaluation of Switch from Satellite Laboratory to Central Laboratory for Testing of Intraoperative Parathyroid Hormone, Pract. Lab. Med. (2020) 22: e00176] [1]

**Specifications Table**

**Table:** 

Subject	Medicine and Dentistry
Specific subject area	Pathology and Medical Technology
Type of data	Supplementary tables Figure
How data were acquired	Retrospective chart and data review from laboratory analysis and surgeries performed at an academic medical center
Data format	Raw and Analyzed
Parameters for data collection	Retrospective data was obtained from the electronic medical record (Epic, Inc.) covering the time period from May 1, 2009 through February 21, 2019. The study had approval as a retrospective study from the University of Iowa Institutional Review Board.
Description of data collection	There were a total of 3,025 intraoperative PTH measurements on 897 unique patients who underwent parathyroidectomy. The switch in testing method from near point-of-care to central laboratory occurred on August 1, 2014. The near point-of-care data encompassed 1,787 tests on 514 unique patients (375 female, 139 male). The central laboratory data included 1,238 tests on 383 unique patients (282 female, 101 male). Data includes patient age, gender, location of surgery (main operating rooms vs. ambulatory surgery center), incision to close time for surgery, and operation start to end times. For the central laboratory data, additional data are provided for the intraoperative PTH turnaround time (TAT) including: sample collected to received by laboratory time, sample received to result verified time, and total TAT. Intraoperative PTH turnaround time was not available for the near point-of-care data as this was not routinely captured. Intraoperative PTH measurements were determined by the same Roche Diagnostics PTH immunoassay at both testing locations, although the near point-of-care testing used a different analyzer than used in the central laboratory.
Data source location	Iowa City, Iowa, United States of America
Data accessibility	Raw data are available in this article as a figure and 2 Supplementary files.
Related research article	Author's name Denise Jacob, Geeta Lal, Dena R. Voss, Tami Bebber, Scott R. Davis, Jeff Kulhavy, Sonia L. Sugg, Anna E. Merrill, Matthew D. Krasowski Title Evaluation of Switch from Satellite Laboratory to Central Laboratory for Testing of Intraoperative Parathyroid Hormone Journal Pract Lab Med 22: e00176. DOI https://doi.org/10.1016/j.plabm.2020.e00176

## Value of the Data

• The data provided is of value as intraoperative PTH measurements present multiple practical challenges.
• Clinicians, other researchers, or personnel in clinical laboratories might find this data useful as a reference for comparison.
• There are limited published data sets that include intraoperative PTH turnaround time.
• The data is of value as there is very limited published data on impact of intraoperative PTH testing on the length of parathyroidectomy surgeries.
• The data provide information for 3025 measurements in 897 unique patients undergoing parathyroidectomy.

## 1 Data Description

**Fig. 1:**
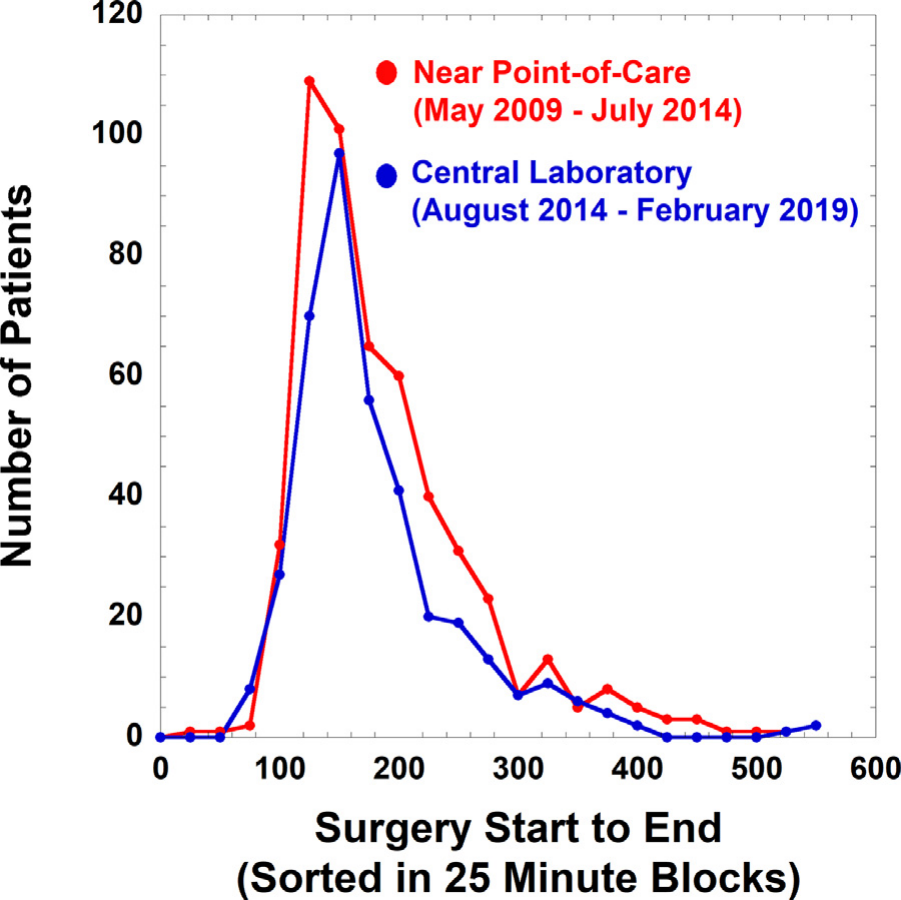
Operation start to end times for parathyroidectomies performed before and after switch from near point-of-care (satellite laboratory, *n* = 514 surgeries, red symbols and lines) to central laboratory (*n* = 383 surgeries, blue symbols and lines) intraoperative PTH testing. The times are grouped in 25 minute blocks.

In this retrospective study, we assembled data on 3025 intraoperative PTH measurements on 897 unique patients who had parathyroid surgery. There are many practical challenges that impact TAT for intraoperative PTH [2], [3], [4], [5], [6], [7], [8], [9], [10]. These include geographic location of operating rooms, transport of samples to testing location, analysis time, and method of reporting of results to the surgical team. Fig. 1 shows operation start and end times for parathyroidectomies performed before and after switch from near point-of-care to central laboratory measurement of intraoperative PTH. The related research article [1] shows plots for incision to close times, a measure of surgery time less affected by other factors unrelated to intraoperative PTH measurement. The raw data are included in Supplementary file 1 (satellite laboratory performing near point-of-care intraoperative PTH testing) and Supplementary file 2 (central laboratory performing testing).

• Supplementary file 1: Data for 1787 intraoperative PTH measurements on 514 unique patients (375 female, 139 male) undergoing parathyroidectomies in which intraoperative PTH was monitored by near point-of-care testing. Specific data fields include: test type (baseline intraoperative PTH measurement versus measurements after gland removal), age in years, birth sex, location of surgery (main operating room versus more distantly located ambulatory surgery center), incision to close time (mins), and operating start to end times (mins).
• Supplementary file 2: Data for 1238 intraoperative PTH measurements on 383 unique patients (282 female, 101 male) undergoing parathyroidectomies in which intraoperative PTH testing was performed in the central laboratory. Specific data fields include: test type (baseline intraoperative PTH measurement versus measurements after gland removal), specimen collect to received in laboratory time (mins), specimen received to result verified time (mins), total TAT (mins), age in years, birth sex, location of surgery (main operating room versus ambulatory surgery center), incision to close time (mins), and operating start to end times (mins).

## 2 Experimental Design, Materials, and Methods

The data were collected as part of a retrospective study approved by the University of Iowa Institutional Review Board (protocol # 201903764) covering the time frame from May 1, 2009 to February 21, 2019. The electronic health record (EHR) for the institution is Epic (Epic, Inc., Madison, WI). Epic Reporting Workbench (RWB), a reporting tool within the electronic medical record, was used to capture all intraoperative PTH orders in the retrospective time period [11]. Data fields that were accessible in RWB further extracted parathyroidectomy incision and close times and operation start to end times for all parathyroidectomies associated with intraoperative PTH measurements. For the data where intraoperative PTH was measured in the central laboratory, additional data fields were pulled to include specimen collection time, specimen received in central laboratory time, and time of verified result in the electronic medical record. Intraoperative PTH TAT was not available for the near point-of-care data as this was not routinely captured.

## Declaration of Competing Interest

The authors declare that they have no known competing financial interests or personal relationships that could have appeared to influence the work reported in this paper.

## Appendix Supplementary materials

Image, application 1
